# Sex Differences in Cardiovascular Disease Risk by Socioeconomic Status (SES) of Workers Using National Health Information Database

**DOI:** 10.3390/ijerph17062047

**Published:** 2020-03-19

**Authors:** Hosihn Ryu, Jihyun Moon, Jiyeon Jung

**Affiliations:** College of Nursing, Korea University, Seoul 02841, Korea; hosihn@korea.ac.kr (H.R.); hepburn86@korea.ac.kr (J.J.)

**Keywords:** worker, sex difference, socioeconomic status, health behavior, cardiovascular disease, risk

## Abstract

The socioeconomic status (SES) and health behaviors of workers are associated with the risks of developing obesity, diabetes, hypertension, hyperlipidemia, and other cardiovascular diseases. Herein, we investigated the factors influencing cardiovascular disease (CVD) risk based on the SES of male and female workers. This cross-sectional analysis used the National Health Information Database to assess the associations between gender, SES (income level, residential area), health behaviors, and CVD-related health status of workers, through multinomial logistic regression. Upon analysis of a large volume of data on workers during 2016, the smoking and drinking trends of male and female workers were found to differ, causing different odds ratio (OR) tendencies of the CVD risk. Also, while for male workers, higher ORs of obesity or abdominal obesity were associated with higher incomes or residence in metropolitan cities, for female workers, they were associated with lower incomes or residence in rural areas. Additionally, among the factors influencing CVD risk, lower income and residence in rural areas were associated with higher CVD risk for male and female workers. The study findings imply the importance of developing gender-customized intervention programs to prevent CVD, due to gender-specific associations between CVD-related health status and health behaviors according to SES.

## 1. Introduction

According to the national health statistics for 2018, 53.2% (about 27.58 million) of the entire Korean population is economically active, and 96.1% of this active population are involved in labor activities [1]. According to the national health statistical yearbook, the prevalence of obesity among Korean adults older than 30 has reached 37.2%, while the prevalence of hypertension, diabetes, hypercholesterolemia, and hypertriglyceridemia has reached 33.5%, 13.0%, 22.1%, and 17.5%, respectively [2]. Obesity, diabetes, hypertension, and hyperlipidemia are important risk factors for cardiovascular diseases (CVD), which contribute to the mortality rate globally [3,4]. Statistics for 2018 showed that 39.0% of the workers who died from disease in Korea had cardio-cerebrovascular diseases, a percentage that has increased over the last few years [5]. In addition, the worker group tended to practice an unhealthier lifestyle than typical adults [6] and had a higher risk of developing cardiovascular disease [7]. 

Socioeconomic status (SES) is reported to have a significant influence on the risk of cardiovascular diseases in adults [8,9,10,11]. SES affects the incidence of cardiovascular diseases through its effect on health status [8]. However, its effect on CVD-related health status varies by gender, and different aspects of SES have varying degrees of impact on health status between men and women [9,11]. These different aspects of SES affect the incidence of cardiovascular diseases in a complex manner. In particular, income level and education level are associated with obesity, abdominal obesity, hypertension, and diabetes [11,12], while residential area is associated with obesity, abdominal obesity, and diabetes [9,10]. Numerous studies have been conducted to elucidate the relationship between SES and CVD-related health status, such as obesity, hypertension, and diabetes, among normal adults; however, studies on the worker group, which comprises a majority of the adult population, are insufficient.

In Korea, the National Health Insurance System (NHIS) was established in 1989 to provide health insurance cover to the entire population [13]; thus, all data on medical treatment are saved in the NHIS database [14]. Small differences that are significant can also be statistically identified in this database as this data source contains all individuals covered by the NHIS. Thus, the NHIS can be considered a large data depository from which, compared to a small population dataset, more accurate results featuring a smaller margin of error can be obtained. These data are useful when conducting research that requires the basic characteristics of individuals as they contain the personal characteristics of the insured, such as income and residential area.

In this study, the database provided by the NHIS, the National Health Insurance Service-National Health Information Database (NHIS-NHID), was employed to verify the association between the SES of male and female workers in Korea and their CVD-related health statuses. The findings of this study will contribute to the production of basic statistics with large volumes of data through an analysis of the factors influencing CVD risk in male and female workers. 

## 2. Materials and Methods 

### 2.1. Data Sources

The NHIS of Korea provides health insurance cover to the entire population of the country [13] and allows an insured worker and his or her dependent to receive a free annual or biannual health examination [15]. Based on the NHIS, the national health information database has a large volume of data, including 1.3 trillion pieces of information on the qualifications, premiums, health examination results, and treatment records of the entire population [16]. The NHIS-NHID is the dataset prepared by extracting, summarizing, and processing the health insurance information collected, possessed, and managed by the NHIS for study purposes, while ensuring that personal information cannot be identified [16]. The NHIS-NHID consists of the qualification database, the health examination database, and the claim database [15]. The qualification database includes gender differentiated into male and female, age, premium, and residential area. The premium is defined as the amount paid thus far by the insured worker, and it is categorized into one of 20 quintiles. The residential area is categorized into codes representing the area of residence of the insured. The health examination database contains data from a regular health examination and the accompanying health questionnaire. The regular health examination records both the results of anthropometric measurements, including height, weight, waist circumference (WC), systolic blood pressure (SBP), and diastolic blood pressure (DBP), and the results of a blood test including fasting glucose (FG), total cholesterol (TC), triglyceride (TG), high-density lipoprotein-cholesterol (HDL-C), and low-density lipoprotein-cholesterol (LDL-C). Before the health examination, a health questionnaire must be filled out. This survey contains questions on health behaviors, such as smoking, drinking, and physical activity. The survey requests current smoking status (No; Yes, in the past; Yes, still smoking), frequency of drinking per week (0–7 days/week), amount drunk daily (glasses), and the frequency of walking, moderate exercise, and vigorous exercise (0–7 days/week).

### 2.2. Study Population and Design

**Figure 1:**
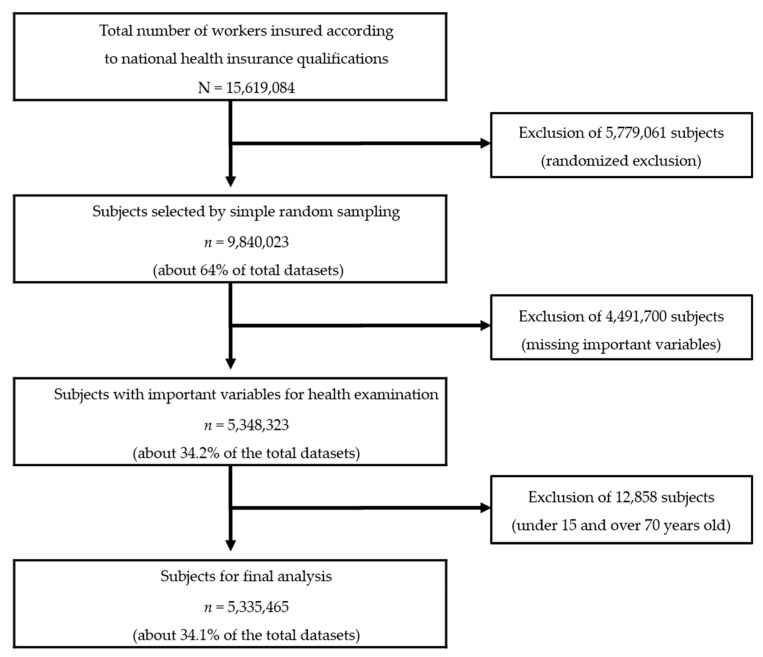
Flow diagram showing study sample and sampling method. N was the total number of the insured workers and n was the number of subjects selected according to the sampling process.

In this population-based nationwide study, instead of using the entire dataset on the insured workers (GAIBJA_TYPE_code 5), simple random sampling, which can be used to obtain generalized results, was applied to obtain the data of 9,840,023 individuals (about 64% of the total dataset) according to the data management manual for NHIS internal data. Subjects with missing health examination records (4,491,700) and those under 15 not involved in labor activities [1] or older than 70 years (12,858) were excluded, leaving a total of 5,335,465 subjects (males: 3,301,052; females: 2,034,413) for analysis (Figure 1). 

This study was conducted following approval from the Institutional Review Board (KU-IRB-18-EX-69-A-1) for the protection of the rights and interests of subjects.

### 2.3. General Characteristics and Socioeconomic Status

The general characteristics of the subjects included gender and age. Age was categorized as: under 30 (15–29), 30s (30–39), 40s (40–49), 50s (50–59), or 60s (60–69). 

As SES variables, only residential area and income were considered. The residential areas of the subjects were classified into “metropolitan city,” meaning the urbanized area in central cities, “small or medium city,” meaning urbanized areas outside central cities, or “rural area,” meaning not urbanized [17]. For the income level of a worker, the vigintiles of the premiums paid by the insured workers were used. The premium was calculated by multiplying the monthly remuneration by the health insurance rate [18], where the monthly remuneration is obtained by taking the salary received from the same place of business for the year and dividing this figure by the number of months worked. This value can be used to identify the incomes of the insured workers. In this study, the premium, initially categorized into vigintiles, was further consolidated into quintiles of increasing income: 1Q, 2Q, 3Q, 4Q, and 5Q. 

### 2.4. Health Behaviors

Smoking, drinking, and physical activity were analyzed to elucidate their impact as health behaviors. For smoking, the subjects were classified as non-smokers, past smokers, and current smokers. For drinking, daily alcohol consumption was approximately calculated by multiplying the number of drinking days per week by the daily drinking amount for the past year, and assuming that one glass of drink contains 10 g of alcohol. Furthermore, light drinkers (≤15 g/day), moderate drinkers (15.01–30 g/day), and heavy drinkers (>30 g/day) were defined according to a previous study [19]. For physical activity, the weekly frequency (5–7, 3–4, 1–2, none) of ≥30 min walks, moderate exercising, and vigorous exercising were recorded [20]. 

### 2.5. CVD-Related Health Status 

For CVD-related health status, the CVD risk indicators of body mass index (BMI), WC, blood pressure (BP), FG, TC, TG, HDL-C and LDL-C, as well as calculated CVD risk were considered. BMI was calculated by dividing weight (kg) by height squared (m^2^). According to the World Health Organization’s (WHO) Asian-specific criteria [21], BMI was categorized into below 23 kg/m^2^ representing normal (including underweight), 23.00 to 24.99 kg/m^2^ representing overweight, and 25.00 kg/m^2^ representing obese [22]. A WC of ≥90 cm for men or ≥85 cm for women was considered to indicate abdominal obesity [23]. BP, including systolic blood pressure (SBP) and diastolic blood pressure (DBP), was classified into normal (<120/80 mmHg), borderline (≥120/80, <140/90 mmHg), and hypertension (≥140/90 mmHg), following the 2018 guidelines of the Korean Society of Hypertension [24]. FG was classified into normal (<100 mg/dL), prediabetes (100–125 mg/dL), and high (≥126 mg/dL), according to the American Diabetes Association (ADA) criteria [25]. For TC, TG, HDL-C, and LDL-C, the National Cholesterol Education Program (NCEP)—Adult Treatment Panel (ATP) III criteria [26] were considered. High TC, high TG, low HDL-C, and high LDL-C were considered as TC ≥ 240 mg/dL, TG ≥ 200 mg/dL, HDL-C < 40 mg/dL, and LDL-C ≥ 160 mg/dL, respectively. CVD risk by gender was computed according to the Framingham 10-year cardiovascular disease risk equation, and age, TC, HDL-C, SBP, smoking, and diabetes were each assigned a weight towards the total score of CVD risk percentages (low, <10%, intermediate, 10%–20%, high, >20%) [27].

### 2.6. Statistical Analysis

Data were analyzed using Statistical Analysis System (SAS) version 9.2 (SAS Inc., Cary, NC, USA). Briefly, descriptive statistics of frequency, percentage, average, and standard deviation (SD) were performed to examine the distribution and level of SES, health behavior, and health status across the subjects. The t-test and χ^2^-test were performed to examine the differences in health behavior and health status by gender according to SES. To derive the odds ratio (OR) of CVD-related health status, multinomial logistic regression was conducted for the following statistically significant variables: age, SES (income and residential area), smoking, drinking, and physical activity (walking, moderate exercise, vigorous exercise). Furthermore, to identify the factors influencing CVD risk, low-risk subjects were used as a reference while high- and moderate-risk subjects were grouped together [28]. For variables, including age, SES (income, residential area), smoking, drinking, physical activity (walking, moderate exercise, vigorous exercise), BMI, WC, BP, FG, TC, TG, HDL-C, and LDL-C, that were statistically significant according to descriptive statistics analysis and risk analysis, multinomial logistic regression was conducted in phases using a regression model. The significance level used for all tests in this study was 0.05. 

## 3. Results

### 3.1. Socioeconomic Status by Gender

Of the 5,335,465 subjects, 3,301,052 (61.8%) were males and 2,034,413 (38.2%) were females (Table 1). Most male and female workers were in their 40s and resided in a metropolitan city. With respect to income, the two highest quintiles for men were 5Q (29.00%) and 4Q (27.21%) while those for women were 2Q (28.93%) and 1Q (27.57%). 

### 3.2. Health Behavior by Age, Income, and Residential Area

When health behavior was compared by age (Table 2), the portion of subjects who walked 5–7 days per week was the highest for all age groups; however, the proportion of the subjects who did not engage in moderate or vigorous exercises (none) was the highest for both male and female workers. In addition, for male workers, the portion of current smokers and heavy drinkers increased up to the 40s (in the 40s, current smoker, 46.29%, heavy drinker, 58.19%) and decreased from the 50s. For female workers, the proportion was found to only decrease with age.

When health behavior was compared by income (Table 3), the proportion of current smokers was lowest for both male (33.87%) and female (1.29%) workers in 5Q, and the proportion of subjects who did not walk or engage in moderate or vigorous exercises (none) was highest for both male (walking, 25.10%, moderate exercise, 49.00%, vigorous exercise, 52.95%) and female (walking, 22.69%, moderate exercise, 52.96%, vigorous exercise, 62.96%) workers in 1Q. Conversely, as male income increased, there was an increase in the proportion of heavy drinkers. 

Furthermore, when health behavior was compared by residential area (Table 4), for both male and female workers, the rural area was identified as having the highest proportion of subjects who did not walk or engage in moderate or vigorous exercise (none) (for male workers walking, 24.39%, moderate exercise, 42.08%, vigorous exercise, 44.64%; for female workers walking, 29.66%, moderate exercise, 55.25%, vigorous exercise, 63.56%). In all residential areas, for men, the proportion of current smokers (metropolitan city, 40.84%, small or medium city, 41.64%, rural area, 42.40%) or heavy drinkers (metropolitan city, 53.78%, small or medium city, 55.58%, rural area, 52.68%) was high, but for women, the proportion of non-smokers (metropolitan city, 93.96%, small or medium city, 96.09%, rural area, 96.44%) and light drinkers (metropolitan city, 67.51%, small or medium city, 69.84%, rural area, 73.33%) was high. 

### 3.3. CVD-Related Health Status by Age, Income, and Residential Area

When CVD-related health statuses of male and female workers were compared by age (Table 5), the portion of subjects with low CVD risk was found to gradually decrease with age for both men (30s, 99.86%, 40s, 98.62%, 50s, 95.72%, 60s, 91.36%) and women (30s, 99.97%, 40s, 99.67%, 50s, 98.27%, 60s, 94.71%). In more detail, as age increased in men, so did the prevalence of hypertension and high FG, while the occurrence of obesity and abdominal obesity were the highest for men in their 30s (obesity, 47.58%, abdominal obesity, 27.18%). On the other hand, as age increased in women, the prevalence of all four factors increased.

When CVD-related health statuses of male and female workers were compared by income (Table 6), for both men and women, at lower incomes, the proportion of subjects at high CVD risk tended to be higher. Meanwhile, the prevalence of both obesity and abdominal obesity increased for men as income increased, but for women, they were highest in 1Q (obesity, 28.00%, abdominal obesity, 17.17%) and lowest in 4Q (obesity, 15.68%, abdominal obesity, 9.12%).

When CVD-related health statuses of male and female workers were compared by residential area (Table 7), the prevalence rates of obesity, abdominal obesity, hypertension, and high FG were lower for men residing in small or medium cities (obesity, 43.96%, abdominal obesity, 23.18%, hypertension, 11.24%, high FG, 7.24%), but lowest for women residing in metropolitan cities (obesity, 21.75%, abdominal obesity, 12.85%, hypertension, 5.68%, high FG, 2.83%).

### 3.4. Odds Ratio of CVD-Related Health Status by Income and Residential Area

**Figure 2:**
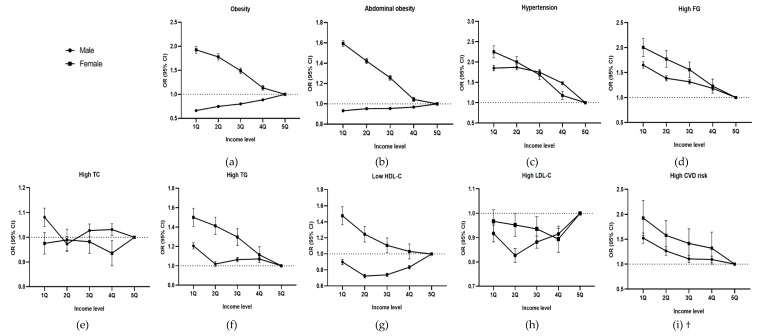
Odds Ratio (OR) of CVD-related health status by income level: (**a**) OR of obesity by income level, (**b**) OR of central obesity by income level, (**c**) OR of hypertension by income level, (**d**) OR of high FG by income level, (**e**) OR of high TC by income level, (**f**) OR of high TG by income level, (**g**) OR of Low HDL-C by income level, (**h**) OR of high LDL-C by income level, and (**i**) OR of high CVD risk by income level. † CVD risk only includes persons older than 30 years and excludes the 58,308 subjects with either a related medical history or currently taking medicines related to CVDs, such as stroke and heart disease. Adjusted for age, region, smoking, alcohol consumption, and frequency of walking, moderate exercise, and vigorous exercise. Abbreviations: OR, odds ratio; CI, confidence interval; Q, Quintile; FG, fasting glucose; TC, total cholesterol; TG, triglyceride; HDL-C, high-density lipoprotein-cholesterol; LDL-C, low-density lipoprotein-cholesterol; CVD, cardiovascular disease. Error bars indicate 95% CI.

When the OR of CVD-related health status was analyzed by income (Figure 2), for men, the ORs of obesity (OR, 0.663; 95% confidence interval (CI), 0.653–0.673) and abdominal obesity (OR, 0.932; 95% CI, 0.921–0.943) were the lowest in 1Q, while the ORs of high FG (OR, 1.650; 95% CI, 1.625–1.676) and high CVD risk (OR, 1.523; 95% CI, 1.482–1.565) were the highest in 1Q and were found to increase as income decreased. For women, the ORs of obesity (OR, 1.926; 95% CI, 1.898–1.955), abdominal obesity (OR, 1.593; 95% CI, 1.566–1.621), hypertension (OR, 2.251; 95% CI, 2.192–2.312), high FG (OR, 2.003; 95% CI, 1.930–2.078), hypertriglyceridemia (OR, 1.500; 95% CI, 1.463–1.539), low HDL-C (OR, 1.475; 95% CI, 1.430–1.521), and high CVD risk (OR, 1.924; 95% CI, 1.787–2.071) all were the highest in 1Q and increased as income decreased. 

**Figure 3:**
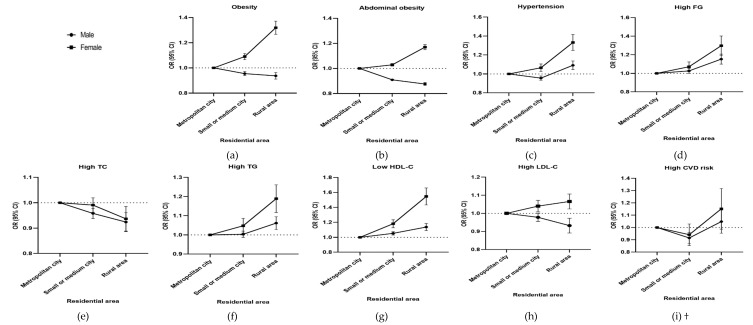
Odds Ratio (OR) of CVD-related health status by residential area. (**a**) OR of obesity by residential area, (**b**) OR of abdominal obesity by residential area, (**c**) OR of hypertension by residential area, (**d**) OR of high FG by residential area, (**e**) OR of high TC by residential area, (f) OR of high TG by residential area, (**g**) OR of low HDL-C by residential area, (**h**) OR of high LDL-C by residential area, and (**i**) OR of high CVD risk by residential area. † CVD risk only includes persons older than 30 years and excludes the 58,308 subjects with either a related medical history or currently taking medicines related to CVDs, such as stroke and heart disease. Adjusted for age, income level, smoking, alcohol consumption, and frequency of walking, moderate exercise, and vigorous exercise. Abbreviations: OR, odds ratio; CI, confidence interval; FG, fasting glucose; TC, total cholesterol; TG, triglyceride; HDL-C, high density lipoprotein-cholesterol; LDL-C, low density lipoprotein-cholesterol; CVD, cardiovascular disease. Error bars indicate 95% CI.

When the OR of CVD-related health status was analyzed according to residential area (Figure 3), the ORs of obesity (for male workers OR, 0.939; 95% CI, 0.926–0.947; for female workers OR, 1.319; 95% CI, 1.298–1.340), abdominal obesity (for male workers OR, 0.874; 95% CI, 0.865–0.888; for female workers OR, 1.169; 95% CI, 1.150–1.190), and high LDL-C (for male workers OR, 0.932; 95% CI, 0.916–0.949; for female workers OR, 1.068; 95% CI, 1.048–1.081) were the lowest for male workers, but highest for female workers residing in rural areas. 

### 3.5. Factors Influencing CVD Risk 

When the factors influencing CVD risk were analyzed (Table 8), we found that for both male and female workers, as age increased and income decreased, the OR of CVD risk increased for obesity, abdominal obesity, hypertension, high FG, hypercholesterolemia, hypertriglyceridemia, low HDL-C, high LDL-C, when subjects resided in a rural area, were current smokers, engaged in walking 3–4 times per week or less, engaged in moderate exercise 1–2 times per week or less, or did not engage in vigorous exercise. There was a marked gender-specific difference. For male workers, the OR of CVD risk was lower for moderate drinkers (OR, 0.828; 95% CI, 0.805–0.852) than light drinkers, but higher for heavy drinkers (OR, 1.298; 95% CI, 1.273 - 1.323); however, for female workers, a decrease in the OR was found for moderate drinkers (OR, 0.668; 95% CI, 0.630–0.708) and heavy drinkers (OR, 0.914; 95% CI, 0.857–0.975).

## 4. Discussion

In the present study, we investigated the associations between SES and CVD-related health status for male and female workers. When comparing the associations for male and female workers, both differences and similarities were observed. The main observations follow. First, the smoking and drinking tendencies varied between male and female workers. Second, while the OR of obesity and abdominal obesity was higher for men with higher incomes or men residing in metropolitan cities, conversely, it was higher for lower income women or women residing in rural areas. Third, the prevalence of belonging to the prehypertension group and the prediabetes group was higher for both male and female workers than all adults (the entire population over 30 years old). In addition, for both male and female workers, as age increased or income decreased, the OR of CVD risk increased for obesity, abdominal obesity, hypertension, high FG, hypercholesterolemia, hypertriglyceridemia, low HDL-C, high LDL-C, when subjects resided in a rural area, were current smokers, engaged in walking 3–4 times per week or less, did moderate exercise 1–2 times per week or less, or did not engage in vigorous exercise. 

Previous studies based on large volumes of data, which could be compared to the findings of this study, focused on all adults alone [29,30] rather than on workers. However, some studies have been conducted among workers in a particular region or at a particular place of business.

Herein, the trends of health behavior and CVD-related health status were found to vary in male workers and female workers, and this was the major finding of the present study. 

In a study that analyzed the smoking and drinking behavior of workers using the Korean Working Conditions Survey (KWCS) [31], the current smoking rate of men was found to be high among teens and middle-aged men; however, a constant decrease was found in women with increase in age, supporting the findings of this study. The period when an individual quits smoking and the amount of smoking performed before an individual quits are the major factors affecting the decrease in CVD risk [32]. The presence of a disease, such as diabetes or hypertension, also affects the decrease in the CVD risk for men [32]; however, overall smoking causes a higher CVD risk for women than men [33]. Based on such results, unlike for women, the OR of CVD risk in male workers who were previous smokers decreased in this study, which may be due to a combination of the period when an individual quits smoking, the amount of smoking performed before quitting, and their health condition. Therefore, besides encouraging smoking cessation programs for current smokers, establishing smoking cessation continuing education programs for men and smoking prevention programs for women is also worthwhile.

Lee and Jeon [31] reported that excessive drinking (≥16 days per month) is highest among male workers in their 40s and 30s and female workers in their 20s. However, this tendency decreased with age for females, similar to the findings of this study. Such a finding also corresponds to the results of a study reporting that the lowest occurrence of cardiovascular events is at a light-to-moderate drinking level [34]. On the other hand, Corrao et al. [35] reported that the maximum cardioprotective effect of alcohol consumption is 72 g/day, and that an alcohol consumption of ≥89 g/day increased the risk of developing CVD. Hence, unlike the heavy drinking identified among male workers, the OR of CVD risk of female workers decreased as alcohol consumption in women is generally at the level associated with cardioprotective effects. Therefore, the OR of CVD risk must be examined in future studies by dividing alcohol consumption at a value closer to 72 g/day.

These differences in health behavior not only affect health status but also SES, and regular smoking and drinking behaviors cause negative effects on SES, especially income level [31]; hence, the difference in health behavior between and male and female workers was found to affect CVD-related health status according to SES. Examining the association between income and obesity or abdominal obesity, Gamlath et al. [11] reported that as the income of workers in Sri Lanka increased, the WC and risk of developing obesity increased for male workers but decreased for female workers, aligning with the findings of this study. Due to work-related stress and unhealthy lifestyles, such as frequent drinking and smoking, male workers are more exposed to health risk factors than female workers [36]. Compared to female workers, a higher number of male workers were identified as heavy drinkers, which indicates that excessive drinking in men increases the risk of the development of obesity and abdominal obesity [12]. Thus, the OR of obesity and abdominal obesity is likely to increase with income. 

The correlation between income and dyslipidemia has been previously analyzed among adults in Korea [37]. As income decreased, for men, the risk of developing high LCL-C tended to decrease while for women, the risk of developing high TG and low HDL-C tended to increase, aligning with the findings of our study. Meanwhile, instead of analyzing hypercholesterolemia, hypertriglyceridemia, low HDL-C, and high LDL-C separately, many studies diagnosed an individual with dyslipidemia if he or she had at least one of these conditions. Therefore, hypercholesterolemia, hypertriglyceridemia, low HDL-C, and high LDL-C should be comprehensibly considered to indicate dyslipidemia and analyzed accordingly in future studies.

Meanwhile, in a previous study that investigated the relationship between residential area and CVD-related health status [10], the researchers emphasized that rural areas often lack medical resources, education, and efforts to prevent diseases compared to urban areas, thereby contributing to the lower health status of rural residents compared to city residents. In contrast, the results of our study showed that the risk of developing obesity and abdominal obesity was likely to be highest for male workers residing in metropolitan cities and female workers residing in a rural areas. This difference is because male workers in urban areas tend to overeat and consume less vegetables than female workers [38], which causes a relatively high risk of obesity and abdominal obesity development among men. However, additional studies considering eating habits should be conducted in the future to further detail the close relationship between CVD-related health status and eating habits [39].

Some similarities in CVD-related health status were also found between male and female workers, as discussed below.

First, the prevalence of belonging to a prehypertension group and prediabetes group was significantly higher for male and female workers than normal adults [29,30]. When individuals belonging to a prehypertension group or prediabetes group neglect their health, they are more prone to progress to hypertension or diabetes [40,41], and their risk of CVD development increases [3,42]. However, workers belonging to the prehypertension group or prediabetes group were either unaware of or uninterested in their disease state, and thus had a reduced desire to maintain good health, which leads to insufficient care [41,43]. Thus, workers in the prehypertension group or prediabetes group, as well as those with hypertension or diabetes, should proactively derive strategies to maintain good health. 

Second, as the age of male and female workers increased, a higher CVD risk was expected. In fact, the risk of developing CVD tended to be higher for female workers than for male workers, and a sharp increase was likely to occur after the 50s. After 40 years old, women experience changes in estrogen levels due to menopause, and the aging process causes abdominal obesity and an abnormal lipid profile, ultimately increasing their risk of CVD [44]. Accordingly, age is a crucial factor for both men and women, particularly for women; thus, a customized program should be developed through in-depth analysis of each age group and then applied. 

Third, it was probable that physical activity was the major factor influencing the CVD risk of male and female workers. In particular, the OR of CVD risk was found to increase for workers that did not walk, which implies that increased walking is very important for reducing BMI, WC, SBP, and DBP [28]. In addition to walking, performing moderate or vigorous exercise on a regular basis is also helpful for preventing CVD [45]. Therefore, programs to increase physical activity at workplaces, such as reducing the time spent seated during work hours or using the stairs, should be adopted to promote physical activity. Further, a comprehensive health management program must be created to reduce CVD risk. The present study is significant as it provides a comprehensive description and reports the health examination and medical survey data of male and female workers from the NHID. Importantly, we found that the health status of workers differed from that of normal adults. Our findings could thus be used as a foundation for developing CVD prevention programs for both male and female workers as CVD-related health status owing to SES and the factors influencing CVD risk were found to vary between male and female workers. Nevertheless, limitations may arise when establishing an accurate causal relationship. Because this study was a cross-sectional one which only analyzed a limited portion of the workers insured within a specific period, its ability to accurately identify cause–effect relationships is limited. In addition, there may be a number of other influencing factors not identified in this database, and so the results are not comprehensive. Due to these limitations, special care is required when interpreting the results in terms of the factors influencing CVD risk. To overcome these drawbacks, more studies analyzing the health examination and medical survey data of a cohort are needed to further determine the health status of male and female workers. Also, this study used only Korean data, and analyses of other countries may lead to different results. Therefore, caution is needed when applying or interpreting this paper in the contexts of other countries.

## 5. Conclusions

To the best of our knowledge, this study is the first global attempt to use a large volume of data from a nationwide database to determine the differences and similarities in CVD-related health status between male and female workers owing to SES. Based on the study findings, we identified gender differences in tendencies towards smoking and drinking, and of the OR and prevalence of obesity and abdominal obesity of workers due to SES. Thus, an obesity or abdominal obesity program must be implemented for high-income male workers or male workers residing in a metropolitan city and for low-income female workers or female workers residing in a rural area. As physical activity was identified as the major influential factor of the CVD risk in male and female workers, a workplace-based program that promotes physical activity should be implemented to improve the health behavior to thereby reduce CVD risk in workers. Altogether, the findings of the present study can be employed as a basic foundation for the future development of a workplace-based intervention program for workers.

## Figures and Tables

**Table 1:** Baseline characteristics of this study cases by sex (*n* = 5,335,465).

	Total Workers		Male Workers		Female Workers		*p*-Value
	(*n* = 5,335,465)		(*n* = 3,301,052)		(*n* = 2,034,413)		
	*n* (%)						
Age							
Under 30	724,715	(13.58)	367,212	(10.83)	357,504	(18.05)	<0.001
30s	1,372,326	(25.72)	450,012	(27.94)	922,314	(22.12)	
40s	1,550,794	(29.07)	575,332	(29.55)	975,461	(28.28)	
50s	1,228,316	(23.02)	479,308	(22.69)	749,009	(23.56)	
60s	459,313	(8.61)	162,550	(8.99)	296,765	(7.99)	
Income level							
1 Q	863,264	(16.18)	302,376	(9.16)	560,888	(27.57)	<0.001
2 Q	1,033,537	(19.37)	444,982	(13.48)	588,556	(28.93)	
3 Q	1,111,566	(20.83)	698,172	(21.15)	413,393	(20.32)	
4 Q	1,184,254	(22.20)	898,216	(27.21)	286,038	(14.06)	
5 Q	1,142,845	(21.42)	957,305	(29.00)	185,538	(9.12)	
Residential area							
Metropolitan city	3,720,540	(69.73)	2,272,444	(68.84)	1,448,095	(71.18)	<0.001
Small or medium city	1,310,832	(24.57)	839,458	(25.43)	471,373	(23.17)	
Rural area	304,094	(5.70)	189,150	(5.73)	114,944	(5.65)	

Abbreviations: Q, Quintile. *p*-values were calculated by cross tabulation analysis (chi-squared test) between male workers and female workers.

**Table 2:** Sex differences of health behavior by age.

	Male Workers						Female Workers					
	Under 30	30s	40s	50s	60s	*p*-Value	Under 30	30s	40s	50s	60s	*p*-Value
	%						%					
Smoking												
Non-smoker	45.18	33.58	24.10	26.37	31.42	<0.001	90.52	92.46	95.52	97.55	97.69	<0.001
Past smoker	10.94	20.58	29.61	38.62	43.56		3.56	3.57	1.95	1.01	0.99	
Current smoker	43.88	45.84	46.29	35.01	25.02		5.92	3.97	2.53	1.44	1.32	
Alcohol consumption												
Light drinker	30.49	27.85	27.54	32.47	44.06	<0.001	48.59	62.67	69.27	80.72	89.33	<0.001
Moderate drinker	15.72	16.13	14.27	14.20	18.15		21.48	20.47	18.34	12.24	6.94	
Heavy drinker	53.79	56.02	58.19	53.33	37.79		29.93	16.86	12.39	7.04	3.73	
Walking												
None	16.46	16.82	18.52	20.57	21.18	<0.001	14.47	19.79	21.34	23.46	26.67	<0.001
1–2 times a week	20.10	26.41	28.54	26.28	18.87		23.29	28.08	30.39	21.15	15.96	
3–4 times a week	19.16	20.76	22.94	24.25	24.25		22.03	20.82	21.95	22.55	19.68	
5–7 times a week	44.28	36.01	30.00	28.90	35.70		40.21	31.31	26.32	32.85	37.69	
Moderate exercise												
None	37.57	39.03	38.17	38.69	43.51	<0.001	47.56	50.64	47.08	50.24	59.09	<0.001
1–2 times a week	35.27	38.92	37.74	33.63	25.10		33.90	32.42	29.95	24.39	17.00	
3–4 times a week	16.94	14.72	16.32	18.12	18.67		13.52	12.12	15.40	16.04	12.90	
5–7 times a week	10.22	7.33	7.77	9.56	12.72		5.02	4.82	7.57	9.33	11.01	
Vigorous exercise												
None	40.12	44.18	42.75	41.91	47.95	<0.001	58.35	62.43	57.25	59.16	68.31	<0.001
1–2 times a week	36.16	37.69	36.64	34.71	26.36		28.65	26.2	26.1	22.82	15.38	
3–4 times a week	15.64	12.78	14.55	15.86	15.83		9.92	8.57	11.61	11.97	9.42	
5–7 times a week	8.08	5.35	6.06	7.52	9.86		3.08	2.80	5.04	6.05	6.89	

*p*-values were calculated by cross tabulation analysis (chi-squared test) within each gender.

**Table 3:** Sex differences of health behavior by income.

	Male Workers						Female Workers					
	1Q	2Q	3Q	4Q	5Q	*p*-Value	1Q	2Q	3Q	4Q	5Q	*p*-Value
	%						%					
Smoking												
Non-smoker	31.01	32.81	29.64	28.29	30.30	<0.001	94.65	93.85	93.88	95.45	96.94	<0.001
Past smoker	27.17	24.74	23.76	27.95	35.82		2.21	2.36	2.59	2.29	1.77	
Current smoker	41.82	42.45	46.61	43.76	33.87		3.14	3.79	3.53	2.25	1.29	
Alcohol consumption												
Light drinker	40.11	37.73	32.03	27.27	26.02	<0.001	74.21	67.09	63.37	64.6	71.15	<0.001
Moderate drinker	14.04	15.01	14.75	15.23	16.11		13.94	16.86	19.23	20.23	17.30	
Heavy drinker	45.85	47.26	53.22	57.49	57.87		11.86	16.05	17.40	15.17	11.55	
Walking												
None	25.10	21.92	20.36	18.00	15.03	<0.001	22.69	20.90	19.50	18.83	19.60	<0.001
1–2 times a week	22.71	20.57	24.01	27.56	28.79		20.53	22.52	25.67	28.72	29.29	
3–4 times a week	19.81	20.46	20.20	22.14	25.22		20.74	21.63	21.91	23.37	22.57	
5–7 times a week	32.38	37.05	35.43	32.30	30.96		36.04	34.95	32.92	30.08	28.54	
Moderate exercise												
None	49.00	45.69	42.66	38.44	32.90	<0.001	52.96	49.95	48.00	47.08	46.97	<0.001
1–2 times a week	27.87	29.65	34.22	38.22	39.68		23.93	27.62	31.76	34.01	33.18	
3–4 times a week	13.92	15.26	14.73	15.66	18.79		14.00	14.75	14.18	13.85	14.22	
5–7 times a week	9.21	9.40	8.39	7.68	8.63		9.11	7.68	6.06	5.06	5.63	
Vigorous exercise												
None	52.95	49.66	46.55	42.52	38.32	<0.001	62.96	59.40	58.34	58.56	58.76	<0.001
1–2 times a week	28.16	30.32	34.58	37.88	38.60		21.12	24.68	27.34	28.1	27.12	
3–4 times a week	11.74	12.97	12.59	13.77	16.56		10.20	11.00	10.46	10.14	10.54	
5–7 times a week	7.15	7.05	6.28	5.83	6.52		5.72	4.92	3.86	3.20	3.59	

*p*-values were calculated by cross tabulation analysis (chi-squared test) within each gender.

**Table 4:** Sex differences of health behavior by residential area.

	Male Workers				Female Workers			
	Metropolitan City	Small or Medium City	Rural Area	*p*-Value	Metropolitan City	Small or Medium City	Rural Area	*p*-Value
	%				%			
Smoking								
Non-smoker	29.97	30.20	33.14	<0.001	93.96	96.09	96.44	<0.001
Past smoker	29.19	28.17	24.46		2.60	1.63	1.34	
Current smoker	40.84	41.64	42.40		3.44	2.28	2.22	
Alcohol consumption								
Light drinker	30.45	30.18	33.36	<0.001	67.51	69.84	73.33	<0.001
Moderate drinker	15.77	14.24	13.96		17.69	15.81	13.73	
Heavy drinker	53.78	55.58	52.68		14.81	14.34	12.94	
Walking								
None	17.20	20.80	24.39	<0.001	18.54	25.06	29.66	<0.001
1–2 times a week	25.06	27.31	25.41		23.36	26.22	25.06	
3–4 times a week	22.42	22.41	20.93		21.84	21.54	20.07	
5–7 times a week	35.32	29.48	29.27		36.26	27.18	25.21	
Moderate exercise								
None	38.76	38.74	42.08	<0.001	48.85	50.76	55.25	<0.001
1–2 times a week	36.05	35.61	32.50		29.51	27.78	25.11	
3–4 times a week	16.52	16.74	16.23		14.42	14.23	12.84	
5–7 times a week	8.67	8.91	9.19		7.22	7.23	6.80	
Vigorous exercise								
None	43.31	42.35	44.64	<0.001	59.62	59.98	63.56	<0.001
1–2 times a week	35.76	35.53	32.54		25.39	24.32	22.10	
3–4 times a week	14.38	15.01	15.14		10.53	10.77	9.76	
5–7 times a week	6.55	7.11	7.68		4.46	4.93	4.58	

*p*-values were calculated by cross tabulation analysis (chi-squared test) within each gender.

**Table 5:** Sex differences of cardiovascular disease (CVD)-related health status by age.

	Male Workers						Female Workers					
	Under 30	30s	40s	50s	60s	*p*-Value	Under 30	30s	40s	50s	60s	*p*-Value
	Mean ± SD or %						Mean ± SD or %					
BMI (kg/m^2^)	24.33 ± 3.80	25.11 ± 3.58	24.91 ± 3.20	24.54 ± 2.88	24.28 ± 2.81		21.45 ± 3.58	22.09 ± 3.69	22.98 ± 3.39	23.57 ± 3.11	24.15 ± 3.07
Obesity	37.05	47.58	46.60	42.40	39.26	<0.001	12.99	17.13	23.28	28.72	36.37	<0.001
WC (cm)	82.08 ± 9.52	84.96 ± 9.14	84.83 ± 8.24	84.81 ± 7.60	82.55 ± 7.77		71.37 ± 8.70	75.05 ± 8.09	75.82 ± 8.08	76.91 ± 8.38	79.74 ± 8.27
Abdominal obesity	19.12	27.18	25.75	24.97	23.58	<0.001	7.05	10.76	12.28	16.52	26.18	<0.001
BP (SBP/DBP, mmHg)	120.68 ± 11.68/ 74.61 ± 8.50	122.86 ± 12.40/ 77.17 ± 9.33	123.69 ± 13.15/ 78.64 ± 9.77	125.04 ± 13.63/ 78.96 ± 9.51	127.00 ± 13.87/ 78.08 ± 9.21		110.95 ± 10.97/ 69.26 ± 8.30	111.71 ± 11.91/ 70.29 ± 8.92	116.04 ± 13.45/ 73.00 ± 9.59	120.20 ± 14.15/ 74.94 ± 9.54	124.19 ± 14.30/ 75.65 ± 9.25
Borderline	51.65	54.56	53.8	55.06	56.55	<0.001	23.66	25.67	36.08	45.35	51.71	<0.001
Hypertension	4.88	9.03	13.05	15.31	18.37		1.22	2.34	5.79	9.67	14.61	
FG (mg/dL)	91.90 ± 14.20	96.37 ± 19.35	101.73 ± 25.01	106.46 ± 9.51	108.19 ± 29.27		88.66 ± 11.93	90.66 ± 14.49	94.10 ± 17.18	97.62 ± 19.83	100.52 ± 21.85
Pre-diabetes	17.39	26.43	34.14	38.52	38.91	<0.001	9.11	12.97	20.09	26.83	31.05	<0.001
High FG	1.06	3.01	7.37	12.52	15.37		0.56	1.20	2.67	5.08	7.81	
TG (mg/dL)	116.77 ± 27.25	155.52 ± 38.49	167.02 ± 39.33	154.84 ± 34.21	137.68 ± 32.88		75.23 ± 16.76	86.53 ± 11.11	95.84 ± 14.00	112.10 ± 20.45	119.86 ± 22.19
High TG	10.80	21.69	25.62	21.64	15.96	<0.001	2.01	3.99	5.22	8.56	10.10	<0.001
HDL-C (mg/dL)	55.13 ± 13.99	52.54 ± 14.23	52.17 ± 14.84	52.32 ± 14.56	52.58 ± 15.05		66.93 ± 15.60	64.66 ± 16.23	62.68 ± 17.38	61.24 ± 16.78	58.73 ± 15.41
Low HDL-C	9.10	13.49	14.68	14.98	15.12	<0.001	1.63	2.62	3.53	4.55	6.32	<0.001
CVD risk†											
Low		99.86	98.62	95.72	91.36	<0.001		99.97	99.67	98.27	94.71	<0.001
Moderate		0.14	1.35	3.53	5.86			0.03	0.33	1.65	4.51	
High		0.00	0.03	0.75	2.78			0.00	0.00	0.09	0.78	

*p*-values were calculated by cross tabulation analysis (chi-squared test) within each gender. † only people over 30 years old, and excluding 58,308 subjects with a related medical history or taking medicine related to CVD diseases such as stroke and heart disease. Abbreviations: BMI, body mass index; WC, waist circumference; SBP, systolic blood pressure; DBP, diastolic blood pressure; FG, fasting glucose; TG, triglyceride; HDL-C, high density lipoprotein-cholesterol; CVD, cardiovascular disease.

**Table 6:** Sex differences of CVD-related health status by income.

	Male Workers						Female Workers					
	1Q	2Q	3Q	4Q	5Q	*p*-Value	1Q	2Q	3Q	4Q	5Q	*p*-Value
	Mean ± SD or %						Mean ± SD or %					
BMI (kg/m^2^)	24.59 ± 3.46	24.46 ± 3.53	24.71 ± 3.54	24.87 ± 3.30	24.86 ± 2.94		23.36 ± 3.54	22.92 ± 3.55	22.39 ± 3.54	21.97 ± 3.32	22.25 ± 3.18	
Obesity	40.32	42.79	43.09	45.52	45.64	<0.001	28.00	24.10	19.56	15.68	16.91	<0.001
WC (cm)	84.74 ± 9.11	83.87 ± 9.18	84.23 ± 8.97	84.77 ± 8.39	85.20 ± 7.66		76.39 ± 13.67	75.08 ± 19.88	74.44 ± 32.77	74.28 ± 39.61	74.36 ± 3.85	
Abdominal obesity	23.82	24.20	24.81	25.75	26.08	<0.001	17.17	13.63	11.06	9.12	10.03	<0.001
BP (SBP/DBP, mmHg)	124.74 ± 13.85/ 77.89 ± 9.77	124.37 ± 13.64/ 77.53 ± 0.64	124.05 ± 13.29/ 77.87 ± 9.59	123.92 ± 12.82/ 78.06 ± 9.48	123.01 ± 12.74/ 77.78 ± 9.42		118.57 ± 14.20/ 73.85 ± 9.57	116.72 ± 13.63/ 73.04 ± 0.42	114.36 ± 12.98/ 71.76 ± 9.21	112.38 ± 12.40/ 70.30 ± 9.05	113.07 ± 13.13/ 70.60 ± 9.48	
Borderline	53.94	53.57	54.34	55.27	53.71	<0.001	41.04	37.50	31.81	26.63	28.64	<0.001
Hypertension	15.09	13.73	12.51	11.76	10.85		8.61	6.28	4.29	2.93	3.87	
FG (mg/dL)	104.73 ± 31.23	101.12 ± 26.09	100.01 ± 24.55	100.00 ± 23.35	101.23 ± 22.75		96.11 ± 19.77	94.13 ± 17.63	92 ± 15.86	91.15 ± 14.28	92.19 ± 14.81	
Pre-diabetes	32.83	30.84	30.32	31.04	33.37	<0.001	23.31	20.02	16.48	14.04	15.93	<0.001
High FG	11.58	8.23	6.73	6.41	7.37		4.42	3.13	2.14	1.54	1.89	
TG (mg/dL)	153.91 ± 32.57	144.14 ± 28.33	153.00 ± 28.34	158.04 ± 25.75	154.50 ± 20.74		104.07 ± 28.75	97.15 ± 16.92	90.63 ± 16.88	86.73 ± 15.28	92.08 ± 16.74	
High TG	21.68	18.40	21.01	22.62	21.67	<0.001	7.15	5.83	4.64	3.84	4.38	<0.001
HDL-C (mg/dL)	52.18 ± 14.87	53.46 ± 15.18	53.23 ± 15.72	52.58 ± 14.16	51.90 ± 13.24		61.53 ± 16.89	63.21 ± 17.15	64.37 ± 16.60	64.68 ± 15.68	63.78 ± 15.96	
Low HDL-C	15.69	13.25	13.22	13.66	14.67	<0.001	4.51	3.45	2.78	2.58	3.01	<0.001
CVD risk †												
Low	94.58	96.1	97.97	98.35	97.84	<0.001	98.01	98.84	99.36	99.62	99.51	<0.001
Moderate	3.88	2.87	1.63	1.40	1.90		1.79	1.07	0.60	0.36	0.47	
High	1.54	1.03	0.40	0.25	0.26		0.21	0.10	0.04	0.02	0.02	

*p*-values were calculated by cross tabulation analysis (chi-squared test) within each gender. † only people over 30 years old and excluding 58,308 subjects with a related medical history or taking medicine related to CVD diseases such as stroke and heart disease. Abbreviations: Q, Quintile; BMI, body mass index; WC, waist circumference; BP, blood pressure; SBP, systolic blood pressure; DBP, diastolic blood pressure; FG, fasting glucose; TG, triglyceride; HDL-C, high-density lipoprotein-cholesterol; CVD, cardiovascular disease; SD, standard deviation.

**Table 7:** Sex differences of CVD-related health status by residential area.

	Male Workers				Female Workers			
	Metropolitan City	Small or Medium City	Rural Area	*p*-Value	Metropolitan City	Small or Medium City	Rural Area	*p*-Value
	Mean ± SD or %				Mean ± SD or %			
BMI (kg/m^2^)	24.77 ± 3.29	24.73 ± 3.29	24.76 ± 3.34		22.66 ± 3.51	22.86 ± 3.48	23.22 ± 3.57	
Obesity	44.29	43.96	44.63	<0.001	21.75	23.19	27.12	<0.001
WC (cm)	84.77 ± 8.51	84.28 ± 8.46	84.04 ± 8.71		75.10 ± 7.35	75.04 ± 74	75.92 ± 797	
Abdominal obesity	26.01	23.18	23.74	<0.001	12.85	13.27	15.64	<0.001
BP (SBP/DBP, mmHg)	124.76 ± 13.14/ 77.81 ± 9.59	123.64 ± 12.87/ 77.81 ± 9.29	123.89 ± 13.06/ 77.92 ± 9.30		115.6 ± 13.65/ 72.17 ± 9.50	116.37 ± 13.46/ 72.81 ± 9.31	117.61 ± 13.89/ 73.49 ± 9.43	
Borderline	53.77	55.70	54.68	<0.001	33.82	37.42	39.32	<0.001
Hypertension	12.30	11.24	12.32		5.68	5.84	7.58	
FG (mg/dL)	100.86 ± 14.31	100.63 ± 14.65	101.24 ± 16.11		93.50 ± 10.09	93.96 ± 10.53	95.17 ± 10.32	
Pre-diabetes	31.72	31.31	31.37	<0.001	18.56	19.68	21.62	<0.001
High FG	7.34	7.24	7.99		2.83	3.06	3.99	
TG (mg/dL)	152.83 ± 30.20	153.19 ± 30.41	153.53 ± 30`69		95.10 ± 10.57	96.63 ± 10.41	101.40 ± 10.87	
High TG	21.07	21.29	21.40	<0.001	5.39	5.73	6.82	<0.001
HDL-C (mg/dL)	52.71 ± 8.02	52.62 ± 8.09	52.34 ± 7.79		63.54 ± 8.50	62.81 ± 8.05	61.06 ± 8.77	
Low HDL-C	13.72	14.03	14.72	<0.001	3.23	3.75	4.92	<0.001
CVD risk†								
Low	97.47	97.8	97.28	<0.001	98.88	98.93	98.41	<0.001
Moderate	2.04	1.77	2.15		1.02	0.97	1.42	
High	0.49	0.43	0.57		0.10	0.10	0.17	

*p*-values were calculated by cross tabulation analysis (chi-squared test) within each gender. † only people over 30 years old and excluding 58,308 subjects with a related medical history or taking medicine related to CVD diseases such as stroke and heart disease. Abbreviations: BMI, body mass index; WC, waist circumference; BP, blood pressure; SBP, systolic blood pressure; DBP, diastolic blood pressure; FG, fasting glucose; TG, triglyceride; HDL-C, high-density lipoprotein-cholesterol; CVD, cardiovascular disease; SD, standard deviation.

**Table 8:** Factors influencing CVD risk.

Variables	Male Workers		Female Workers	
	OR	95% CI Wald F	OR	95% CI Wald F
Age				
30s	reference		reference	
40s	9.217	8.702–9.762	10.552	8.846–12.588
50s	23.648	22.352–25.019	50.033	42.107–59.450
60s	36.951	34.877–39.149	125.506	105.526–149.269
SES				
Income				
1Q	1.523	1.482–1.565	1.924	1.787–2.071
2Q	1.265	1.231–1.300	1.576	1.462–1.700
3Q	1.105	1.076–1.135	1.412	1.298–1.535
4Q	1.094	1.067–1.121	1.317	1.194–1.454
5Q	reference		reference	
Residential Area				
Metropolitan city	reference		reference	
Small or medium city	0.915	0.897–0.934	0.941	0.907–0.977
Rural area	1.048	1.010–1.086	1.151	1.086–1.219
Health behavior				
Smoking				
Non-smoker	reference		reference	
Past smoker	0.811	0.792–0.830	1.352	1.188–1.538
Current smoker	1.273	1.247–1.300	1.369	1.226–1.528
Alcohol consumption				
Light drinker	reference		reference	
Moderate drinker	0.828	0.805–0.852	0.668	0.630–0.708
Heavy drinker	1.298	1.273–1.323	0.914	0.857–0.975
Walking	reference		reference	
None	1.722	1.583–1.899	2.003	1.865–1.211
1–2 times a week	1.100	1.086–1.125	1.183	1.137–1.206
3–4 times a week	1.036	1.010–1.062	1.056	1.029–1.083
5–7 times a week	reference		reference	
Moderate exercise				
None	1.051	1.023–1.078	1.044	1.028–1.075
1–2 times a week	1.021	1.005–1.038	1.015	1.004–1.036
3–4 times a week	0.939	0.901–0.979	0.970	0.901–1.045
5–7 times a week	reference		reference	
Vigorous exercise				
None	1.118	1.073–1.166	1.125	1.041–1.216
1–2 times a week	1.009	0.966–1.054	0.974	0.894–1.062
3–4 times a week	1.005	0.960–1.052	0.972	0.886–1.066
5–7 times a week	reference		reference	
CVD-related health status				
BMI				
Normal	reference		reference	
Overweight	1.803	1.762–1.844	1.181	1.160–1.203
Obesity	2.649	2.598–2.701	1.546	1.501–1.592
WC				
Normal	reference		reference	
Abdominal obesity	1.471	1.440–1.503	1.257	1.203– 1.313
BP				
Normal	reference		reference	
Borderline	1.060	1.036–1.085	1.044	1.019–1.070
Hypertension	1.305	1.277–1.334	1.541	1.465–.620
FG				
Normal	reference		reference	
Pre-diabetes	1.481	1.166–1.497	1.491	1.468–.515
High FG	1.851	1.847– 1.897	1.979	1.953–.806
TC				
Normal	reference		reference	
Hypercholesterolemia	1.030	1.008–1.052	1.019	0.988–1.051
TG				
Normal	reference		reference	
Hypertriglyceridemia	1.091	1.073–1.110	1.011	0.961–1.150
HDL-C				
Normal	reference		reference	
Low HDL-C	1.041	1.023–1.061	1.103	1.070–1.144
LDL-C				
Normal	reference		reference	
High LDL-C	1.052	1.035–1.071	1.038	1.010–1.066

Subjects include only people over 30 years old, excluding 58,308 subjects with a related medical history or taking medicine related to CVD disease, such as stroke and heart disease. Abbreviations: OR, odds ratio; CI, confidence interval; Q, Quintile; CVD, cardiovascular disease; BMI, body mass index; WC, waist circumference; BP, blood pressure; FG, fasting glucose; TC, total cholesterol; TG, triglyceride; HDL-C, high-density lipoprotein-cholesterol; LDL-C, low-density lipoprotein-cholesterol. Adjusted for age, income level, residential area, smoking, alcohol consumption, frequency of walking, moderate, and vigorous exercise, BMI, WC, BP, FG, TC, TG, HDL-C, and LDL-C.
